# Asystole after Orthotopic Lung Transplantation: Examining the Interaction of Cardiac Denervation and Dexmedetomidine

**DOI:** 10.1155/2012/203240

**Published:** 2012-10-08

**Authors:** Christopher Allen-John Webb, Paul David Weyker, Brigid Colleen Flynn

**Affiliations:** ^1^Columbia University College of Physicians and Surgeons, Columbia University Medical Center, New York, NY 10032, USA; ^2^Division of Critical Care, Department of Anesthesiology, Columbia University Medical Center, 622 West 168th Street PH 5-505, New York, NY 10032, USA

## Abstract

Dexmedetomidine is an **α**_2_-receptor agonist commonly used for sedation and analgesia in ICU patients. Dexmedetomidine is known to provide sympatholysis and also to have direct atrioventricular and sinoatrial node inhibitory effects. In rare instances, orthotopic lung transplantation has been associated with disruption of autonomic innervation of the heart. The combination of this autonomic disruption and dexmedetomidine may be associated with severe bradycardia and/or asystole. Since orthotopic lung transplant patients with parasympathetic denervation will not respond with increased heart rate to anticholinergic therapy, bradyarrhythmias must be recognized and promptly treated with direct acting beta agonists to avoid asystolic cardiac events.

## 1. Introduction

Bradycardia and asystole in the cardiothoracic intensive care unit (CTICU) setting can be due to a broad range of etiologies. Certain patients may be more predisposed to bradyarrhythmias and/or asystole due to a combination of factors such as the surgical procedure, medications being administered, and preexisting patient comorbidities. We report a case of severe bradycardia leading to asystole after a bilateral orthotopic lung transplantation (OLT) in a patient receiving dexmedetomidine sedation. We consider the possibility that cardiac sympathetic denervation secondary to the OLT in conjunction with atrial-ventricular node conduction delay caused by the dexmedetomidine may have led to the asystolic event.

## 2. Case Description

A 55-year-old woman with sarcoidosis was admitted to the CTICU after receiving a bilateral OLT involving significant tracheal dissection. On postoperative day 1, sedation was weaned, and she was communicating appropriately while intubated and desired to have an epidural catheter placed for pain control prior to extubation. Timing of the epidural for patients on anticoagulation was in accordance with the American Society of Regional Anesthesia and Pain Medicine guidelines [1]. Two hours prior to the procedure, the patient was started on a dexmedetomidine infusion at 0.5 micrograms/kilogram/hour for anxiolysis during the procedure. Since bradycardia is oftentimes associated with large boluses and high-dose infusions of dexmedetomidine, we decided to start the infusion at a lower dose two hours prior to procedure. She remained spontaneously breathing, comfortable, and cooperative. Her hemodynamics remained unchanged during the initiation of the dexmedetomidine infusion.

With her assistance and the endotracheal tube remaining in place, she was placed in a sitting position for epidural placement, and 50 micrograms of intravenous fentanyl was given for additional analgesia. Hemodynamic and respiratory stability was maintained with a sinus rhythm of 95 beats per minute, blood pressure of 130/63 mmHg, and SpO_2_ of 95% and was consistent with her vitals prior to initiating the dexmedetomidine infusion. After obtaining the sitting position, her sinus rhythm deteriorated to bradycardia of 30 beats per minute, and she subsequently became hypotensive. She became agitated, ceased following commands, and became unresponsive. The dexmedetomidine infusion was immediately stopped, and one milligram of intravenous atropine was administered for the bradycardia with no effect. The bradycardia continued to decline and deteriorated to an asystolic event confirmed by arterial pressure monitoring, and cardiopulmonary resuscitation (CPR) was initiated. After 3–5 minutes of CPR, an additional 1-milligram bolus of intravenous atropine was administered along with a 1-milligram bolus of intravenous epinephrine, resulting in a heart rate of 130 beats per minute and a systolic blood pressure of 220 mmHg, which rapidly decreased to 130 mmHg. Her hemodynamic profile remained stable, and the epidural placement was aborted. Transesophageal echocardiogram (TEE) failed to show any signs of a pulmonary embolus.

Differential diagnosis for the asystolic arrest included myocardial infarction, pulmonary embolism (PE), relative hypovolemia, a vasovagal event, the Bezold-Jarisch reflex, cardiac sarcoidosis, and direct atrial-ventricular conduction inhibition by dexmedetomidine. Our patient had increased troponin levels; however, these were difficult to interpret due to the CPR efforts and the previous day's lung transplantation operation. Her electrocardiogram remained in sinus rhythm after the resuscitation with no ST abnormalities, and her TEE was unchanged from her baseline, arguing against a saddle embolus or myocardial infarction as the cause of the arrest. While patients with systemic inflammatory diseases are at risk for developing a deep venous thrombosis, this patient was on prophylactic subcutaneous heparin. Cardiac sarcoidosis was also considered; however, this was less likely given that during the preoperative evaluation she denied any previous history of arrhythmias or knowledge of cardiac sarcoidosis. In terms of relative hypovolemia, our patient's fluid balance was negative; however, she was maintaining adequate perfusion pressures with sufficient urine output. Additionally, her ICU bed had previously been positioned in the semirecumbent position with no hemodynamic instability. Unfortunately, she never regained consciousness following the arrest. Computed tomography (CT) of her head and electroencephalogram (EEG) did not demonstrate pathology explaining her comatose state, and she did not meet the criteria for brain death. She remained unresponsive and expired almost a month later from massive gastrointestinal bleeding. On postmortem autopsy, several small pulmonary infarcts consistent with pulmonary emboli were noted. However, the significance and timing of these infarcts could not be established with certainty.

## 3. Discussion

Dexmedetomidine, a D-enantiomer of medetomidine, is a highly selective agonist of G-protein-coupled *α*_2_-adrenergic receptors. These receptors are further categorized into three main subtypes which exist in the periphery (*α*_2*A*_), brain, and spinal cord (*α*_2*B*_, *α*_2*C*_) [2, 3]. Dexmedetomidine is a useful sedative/analgesic commonly used in the ICU for several reasons. It has been shown to decrease time on the ventilator [4], decrease length of ICU stay [3], decrease anxiety, and have opioid-sparing effects due to its analgesic properties [5, 6]. Particularly, in postthoracotomy patients, it has been shown that intravenous dexmedetomidine, in addition to a thoracic epidural bupivicaine infusion, decreases opioid requirements and may decrease the potential for respiratory depression [6]. A significant advantage of dexmedetomidine is its potential attenuation of delirium in ICU patients [6]. This delirium-sparing effect may be due to action on *α*_2_ receptors in the locus ceruleus producing a sedative state similar to physiologic sleep. Lastly, dexmedetomidine is especially beneficial for postsurgical patients in that it preserves the respiratory drive, provides sympatholysis and decreases the inflammatory response [7].

The most common side effects during dexmedetomidine infusion are hypotension and bradycardia [3]. According to the manufacturers of Precedex (Hospira, Inc., Lake Forest, IL), 14% of patients experience bradycardia defined as a heart rate < 40 bpm or >30% decrease from baseline. Other authors have reported an incidence of bradycardia of up to 42% [4]. Bradycardia seen with dexmedetomidine is likely due to sympatholysis in addition to direct inhibitory effects on the sinoatrial (SA) and atrial-ventricular (AV) nodes [8]. This bradycardia usually resolves with cessation of the infusion or treatment with an anticholinergic agent. We believe that dexmedetomidine-induced SA and AV nodal depression proved refractory to anticholinergic medication (atropine) owing to cardiac denervation occurring at the time of lung transplantation.

It is known that during heart transplantation, both sympathetic and parasympathetic denervation occur leading to an increase in basal heart rate as the heart rate and electrical activity of the transplanted heart become entirely dependent on the intrinsic electrical system of the heart rather than relying on the neurological input from the recipient. The milieu of the denervated heart is complicated by the fact that while separation of sympathetic and parasympathetic occurs centrally, there is evidence that postganglionic fibers remain functional in these patients [9]. In other words, following cardiac transplantation, the sinus node becomes decentralized rather than totally denervated [10]. Surgical decentralization of the sinus node may be associated with supersensitivity to catecholamine stimulation [10], because *α* and *β* receptors are still present on the heart. The decentralized heart will continue to respond to directly acting catecholamine agents, such as epinephrine, isoproterenol, and dobutamine [11]. However, due to interruption of parasympathetic tone, the heart is not responsive to anticholinergic agents.

However, there is less knowledge of cardiac decentralization following lung transplantation. The operative dissection, especially that surrounding the trachea, likely leads to interruption of sympathetic and parasympathetic pathways to the heart [12]. This was illustrated in a study by Schaefers et al. which demonstrated that lung transplant recipients displayed abnormal responses to carotid sinus massage and Valsalva maneuvers [12]. In fact, half of the patients studied had no response to atropine, similar to what was observed in our patient. Following OLT, it is thought that reinnervation may occur at around 12 months.

While atrial arrhythmias following lung transplantation can occur during the immediate postoperative period, bradycardia is less defined in the literature. Atrial arrhythmias following lung transplantation are commonly due to inflammation or edema at the pulmonary vein left atrial anastomoses [13]. The arrhythmias occur early during the postoperative period and decrease over time [13]. 

Previous case reports have emerged citing dexmedetomidine as a contributing source of bradycardia followed by asystole in various settings [14–16]. In these reports and in our patient, it is likely that a combination of factors contributed to the asystolic arrest. While the use of concomitant sedatives with sympatholytic properties, that is, fentanyl, may have augmented the bradycardia and/or asystole associated with dexmedetomidine, it is likely that cardiac sympathetic decentralization following OLT placed our patient at significant risk for an asystolic event especially when sedated with dexmedetomidine. To our knowledge, only one other case report has described dexmedetomidine-induced asystole in a patient status after double lung transplant [16]. However, the transient bradycardia and asystole in that patient are confounded by the presence of pulmonary hypertension and a preexisting vagal response to coughing which led to hypoxemia [16]. Additionally, treatment with atropine or glycopyrrolate was not required in this patient. Our case is unique given that our patient demonstrated a refractory response to atropine and required epinephrine for management of the asystolic arrest.

 Clinicians must be aware of the possibility of cardiac chronotropic alterations following OLT, similar to that following heart transplantation. Acknowledging the valuable and beneficiary role that dexmedetomidine plays in the management of OLT patients, we would suggest that heightened vigilance and prompt recognition of dysrhythmias when using dexmedetomidine in these patients may prevent a life-threatening event.
